# Symbiotic microalgae and microbes: a new frontier in saline agriculture

**DOI:** 10.3389/fmicb.2025.1540274

**Published:** 2025-04-22

**Authors:** Cheng-Gang Ren, Cun-Cui Kong, Si-Ming Li, Xiao-Jing Wang, Xiao Yu, Yin-Chu Wang, Song Qin, Hong-Li Cui

**Affiliations:** ^1^Key Laboratory of Coastal Biology and Biological Resources Utilization, Yantai Institute of Coastal Zone Research, Chinese Academy of Sciences, Yantai, China; ^2^College of Resources and Environmental Engineering, Ludong University, Yantai, China; ^3^College of Agronomy, Shanxi Agricultural University, Jinzhong, China; ^4^National Basic Science Data Center, Beijing, China; ^5^Academician Workstation of Agricultural High-Tech Industrial Area of the Yellow River Delta, National Center of Technology Innovation for Comprehensive Utilization of Saline-Alkali Land, Dongying, China

**Keywords:** saline agriculture, salt tolerance, alkali stress, synergistic inoculation, algae-bacteria-plant interactions

## Abstract

With the growing human population worldwide, innovative agricultural development is needed to meet food security needs. However, this has inadvertently led to problematic irrigation practices and overuse of agrochemicals. Such practices can exacerbate soil salinization, which prevents plant growth. As a progressively widespread and escalating problem, soil salinization poses a major threat to global food security. Compared with the traditional use of microalgae or microorganisms that act on plant growth, microalgae–microorganism symbiosis has significant advantages in promoting plant growth. Microalgae and microorganisms can work together to provide a wide range of nutrients required by plants, and they exhibit nutrient complementarity, which supports plant growth. Here, the development potential of microalgae–microbial symbiosis for enhancing plant salt tolerance was investigated. Our review demonstrated that the metabolic complementarity between microalgae and microorganisms can enhance plant salt tolerance. The diversity of a microalgae–microorganism symbiotic system can improve ecosystem stability and resistance and reduce the incidence of plant disease under salt stress. These systems produce bioactive substances (e.g., phytohormones) that promote plant growth, which can improve crop yield, and they can improve soil structure by increasing organic matter and improving water storage capacity and soil fertility. Exploiting the synergistic effects between microalgae and beneficial microorganisms has biotechnological applications that offer novel solutions for saline agriculture to mitigate the deleterious effects of soil salinity on plant health and yield. However, there are several implementation challenges, such as allelopathic interactions and autotoxicity. To make microalgae–bacteria consortia economically viable for agricultural applications, optimal strains and species need to be identified and strategies need to be employed to obtain sufficient biomass in a cost-effective manner. By elucidating the synergistic mechanisms, ecological stability, and resource utilization potential of microalgae–microbial symbiotic systems, this review clarifies salt stress responses and promotes the shift of saline–alkali agriculture from single bioremediation to systematic ecological engineering.

## 1 Introduction

The human population is growing worldwide, and solutions to pressing food security issues are urgently needed, driving the need for innovative agricultural development (Ren et al., 2022). However, agricultural expansion has also inadvertently led to problems such as irrational irrigation practices and overuse of agrochemicals, which exacerbate soil salinization (Mustafa et al., 2019; Singh, 2021). This secondary soil salinization, which results from human activities, is also affected by poor drainage and environmental factors (Cuevas et al., 2019).

Soil salinization prevents plant growth by causing soil salt concentrations to exceed the osmotic pressure of plant tissues, which reduces water uptake (Rengasamy, 2010). Additionally, excess soil salinity causes osmotic stress, deficiency of nutrients that can reduce oxidative stress, and results in ion toxicity to plants. Consequently, salinization can negatively impact vegetative growth, germination, and reproductive development of plants, thereby decreasing crop yield (Machado and Serralheiro, 2017; Mustafa et al., 2019). The consequences of soil salinization threaten the ecological balance, agricultural productivity, and the sustainable and rational use of water and land resources (Daliakopoulos et al., 2016; Singh, 2022).

Despite the negative effects of saline and alkaline environments on plant growth and crop yield, plants show some adaptive capacity by having their own response factors that affect gene expression (Verma et al., 2023), and externally through rhizosphere organisms and other factors (Munns and Gilliham, 2015). Therefore, possible strategies to improve salt and alkali tolerance in plants include inoculation with salt- and alkali-tolerant microorganisms and regulating the balance of the microbial community to promote plant growth (Pan et al., 2024).

Microalgae, bacteria, and fungi are beneficial organisms that constitute soil microbial communities, and microalgae–microorganism symbiosis can greatly enhance plant growth in saline conditions (Gonzalez-Gonzalez and De-Bashan, 2023). Furthermore, they are renewable resources that have various applications in agriculture (Alvarez et al., 2021). Microalgae and microorganisms can work together to provide a wide range of nutrients required by plants, providing complementary nutrients, which supports plant growth, and they can alleviate salt stress (Mutale-Joan et al., 2021; Maurya et al., 2024).

Here, the potential of using microalgae–microbial symbiosis to enhance plant salt tolerance and synergize plant growth in saline and alkaline environments is investigated. By elucidating the synergistic mechanisms, ecological stability, and resource utilization potential of microalgae–microbial symbiotic systems, we help elucidate cross-kingdom interactions in salt stress responses and promote the shift of saline–alkali agriculture from single bioremediation to systematic ecological engineering. Compared with existing reviews, we combined synthetic biology, circular economy, and field validation knowledge to offer a solution with both scientific value and prospects for application in sustainable agriculture.

## 2 Advantages of microalgal–microbial symbiosis

Microalgae are members of the microbial community with promising applications (González-González and de-Bashan, 2021). They have been shown to be effective biofertilizers for a wide range of crops, increasing soil fertility and reducing dependence on synthetic fertilizers (Ng et al., 2024), improving plant salt tolerance (Carillo et al., 2020), and providing large amounts of nutrients to support extensive plant cultivation on saline soils (Ergun et al., 2020). Moreover, they have gained interest as a source of biofuel (Hoang et al., 2023).

Plant growth-promoting bacteria (PGPB) are found mainly in the inter-root soil, foliage, and stem surfaces of plants and promote growth by enhancing the plant’s resistance to salt stress (Compant et al., 2010; Mishra et al., 2021). Rhizosphere microorganisms, especially members of phylum Ascomycota and arbuscular mycorrhizal fungi, can positively affect a variety of plant physiological properties, including exchange capacity, stomatal conductance, photosynthetic pigments, proline, and phenolic content, by interacting with the root system of the plant under conditions of salt stress (Alam et al., 2019; Bencherif et al., 2019). These microbial interactions offer new perspectives for promoting plant growth in saline environments.

Compared with the traditional single microalgae or microorganisms acting on plant growth, microalgae–microorganism symbiosis has significant advantages in promoting plant growth. Microalgae and microorganisms can work together to provide a wide range of nutrients required by plants, such as nitrogen, phosphorus, and potassium, and undergo nutrient complementarity. Joint application of microalgae and nitrogen-fixing bacteria can result in both performing more complex tasks than either can complete alone, and the execution of functions that are difficult for or unachievable by individual strains or species; this can even enhance certain processes that have biotechnological applications (Llamas et al., 2023). Croft et al. (2005) found that photosynthetic oxygen produced by microalgae or cyanobacteria is used by bacteria as an electron acceptor for degradation of organic matter. In turn, bacteria can provide microalgae with other micronutrients such as vitamin B, thus providing a selective advantage for microalgae and ultimately promoting plant growth (Croft et al., 2005). The combined effect of the microalgae and microorganisms can stimulate the plant’s defense system and secrete fungal enzymes as well as antibiotics, helping the plant to avoid pests and diseases (Najdenski et al., 2013; Michalak and Chojnacka, 2015; Fuentes et al., 2016). Furthermore, consortia of microalgae and bacteria, including nitrogen-fixing bacteria, have been found to have biotechnological potential, such as for biofuel production, as biofertilizers for agriculture, and for decontamination of wastewater (Zhang et al., 2020; Llamas et al., 2023).

The oxygen and organic matter produced by microalgae photosynthesis provide the microorganisms with an energy and carbon source, which indirectly improves the photosynthetic efficiency of the plant. Ma et al. (2023) showed that *Chlorella* had a positive effect on the growth and photosynthetic characteristics of quinoa under salt stress conditions. Furthermore, plant growth regulators and antibiotic substances produced during the combined microalgae–microbial application could improve plant stability under salt stress, such as by producing polysaccharides and phytohormones (e.g., growth hormones and cytokinins) that can promote plant growth (Michalak and Chojnacka, 2015). Additionally, *Chlamydomonas* produce phytohormones such as indole-3-acetic acid (IAA), which is an essential signaling molecule that controls various aspects of plant development and promotes plant–bacteria symbiosis (Calatrava et al., 2024).

Considerable research progress has been made in the individual application of microalgae, bacteria, and fungi in plant growth (Kang et al., 2021), but research on their joint application is still relatively scarce. To mitigate the range of pressures from secondary soil salinization in the future, it will be necessary to explore the joint application of microalgae–microbial symbiosis in saline agriculture to fully exploit their value.

## 3 Microalgal and microorganism effects on plant growth under salt and alkali stress

Microalgae have a variety of molecular mechanisms to cope with salt and alkali stress, including ion transport, accumulation of anti-osmotic substances, and enhanced antioxidant function (Shetty et al., 2019). The phycosphere is the region around an algal cell that is rich in various compounds, including nutrients and phytohormones such as IAA (Calatrava et al., 2024).

Microbial colonies with the ability to promote plant growth live near the plant root system and can help the plant to re-establish ionic and osmotic homeostasis, reduce cellular damage to the plant in response to stress, and restore growth under salt and alkali stress (Bhattacharyya and Jha, 2012). Some of the known effects of microalgal and microbial species on plant growth under saline and alkaline conditions are shown in Table 1.

**Table 1:** Use of microalgae or microorganisms to promote plant growth under alkali or salt stress conditions.

Microalgae /microorganism species	Crop	Saline–alkali stress	Application mode	Effect	References
*Dunaliella salin* and *Phaeodactylum tricornutum*	Pepper	Salt stress	Root irrigation	Improved pepper germination and root growth	Kapoore et al. (2021)
*Chlorella pyrenoidosa*	*Chenopodium quinoa*	Salt stress	Root irrigation	Increased quinoa yields	Yang et al. (2023)
Microalgae–cyanobacteria extract	Tomato	Salt stress	Root irrigation	5% mixed microalgae–cyanobacteria extract promoted tomato plant growth	Mutale-Joan et al. (2021)
*Enterobacter cloacae* PM23	*Zea mays*	Salt stress	Soaking seed	Promoted corn plant growth	Ali et al. (2022)
*Stenotrophomonas maltophilia* BJ01	*Arachis hypogaea* (Peanut)	Salt stress	Root irrigation	*Promoted peanut plant growth*	Alexander et al. (2020)
*Kosakonia sacchari* MSK1	*Vigna radiata*	Salt stress	Root irrigation	Increased *Vigna radiata* production	Shahid et al. (2021)
*Sphingobacterium* BHU-AV3	Tomato	Salt stress	Root irrigation	Reduced tomato plant senescence	Vaishnav et al. (2020)
*Pseudomonas fluorescens*, *Bacillus pumilus*, and *Exiguobacterium aurantiacum* (alone and in consortium)	Wheat	Salt stress	Root irrigation	Promoted wheat growth	Nawaz et al. (2020)
*Aneurinibacillus aneurinilyticus* and *Paenibacillus* sp. ACC02 and ACC06	*Phaseolus vulgaris*	Salt stress	Soaking seed	Promoted growth of string bean roots and sprouts	Gupta and Pandey (2019)
*B. pumilus* FAB10	Wheat	Salt stress	Soaking seed	*Improved stress response of wheat under salt stress conditions*	Ansari et al. (2019)
Pinophilic *Talaromyces pinophilus*	Rice	Salt stress	Root irrigation	Increased rice plant length	Khalmuratova et al. (2015)
Mycorrhizae of *Rhizophagus irregularis*	*Colocasia esculenta* L. Schott cv. Criolla	Salt stress	Roots inoculation	Spore colonization suggested that symbiotic associations had a positive effect on plant development with or without salt stress	Baltazar-Bernal et al. (2022)
*Bacillus* sp. Jrh14-10	*Arabidopsis* seedlings	Alkali stress	Root irrigation	Promoted growth and development of *Arabidopsis* seedlings	Guo et al. (2023)
*Bacillus* sp. NBRI YN4.4	*Zea mays*	Alkali stress	Soaking seed	Protective effect on *Zea mays* plant growth under osmotic stress	Dixit et al. (2020)
*Bacillus licheniformis* (strain SA03)	Chrysanthemum	Salt–alkali stress	Root irrigation	Increased survival of *Chrysanthemum* plants and reduced sodium concentration in inoculated plants	Zhou et al. (2017)
*Streptomyces paradoxus* D2-8	Soybean	Salt–alkali stress	Soaking seed	Promoted soybean seedling growth and increases yield	Gao et al. (2022)
Halophilic, alkaliphilic, and haloalkaliphilic strains	Wheat	Salt–alkali stress	Synthetic soil microbial communities	Promoted root growth and higher yields in wheat	Torbaghan et al. (2017)
*Chlamydomonas* sp.	‘Camarosa’ strawberry (*Fragaria* × *ananassa* Duch)	Salt stress	Foliar spraying	Enhanced growth, yield, and physiological traits	Jalalian et al. (2024)

Plant hormones play a crucial role in enhancing the ability of plants to cope with salt–alkali stress environments through the consortium of microalgae and microbes. These hormones, such as auxins, gibberellins, cytokinins, and abscisic acid, are involved in various physiological processes, including cell elongation, root development, stomatal regulation, and stress response. For example, auxins promote root growth and development, thus enhancing the plant’s ability to absorb water and nutrients (Palacios et al., 2016); gibberellins are involved in cell elongation and promote plant growth, and can enhance growth under salt stress (Sahoo et al., 2014); cytokinins are involved in cell division and differentiation and can help plants maintain their growth and productivity under saline conditions (Ansari et al., 2019); and abscisic acid regulates stomatal closure, osmotic adjustment, and the expression of stress-related genes (Barnawal et al., 2017).

The cooperation between microalgae and microbes enhances plant salt tolerance through multiple mechanisms, including nutrient acquisition and metabolic complementarity (Maurya et al., 2024); production of extracellular compounds that can improve soil structure, enhance water retention, and protect plants from salt stress (Gheda and Ahmed, 2015); modulation of plant stress response by producing stress-related compounds and enzymes, such as antioxidants, which protect plants from oxidative damage caused by salt stress (Bhagat et al., 2021); enhancement of plant growth and development by promoting root elongation, increasing biomass, and improving photosynthetic efficiency (Marulanda et al., 2010); and the improvement of soil health by increasing organic matter content, enhancing soil aggregation, and promoting nutrient cycling (Rocha et al., 2020). These mechanisms provide a comprehensive approach that can be harnessed to improve plant productivity in saline–alkali environments.

## 4 Mechanisms of interaction between microalgae and microbial co-cultures

Current applications of microalgal biotechnology essentially require large amounts of microalgal biomass (Rizwan et al., 2018). This increases the risk of contamination of microalgae with bacteria or fungi, which may lead to a decrease in microalgal biomass or even mass mortality in the culture (Bui-Xuan et al., 2022). However, it has been demonstrated that the combined application of microalgae and microorganisms can positively affect plant growth while retaining normal growth of the microalgae and microorganisms (Dao et al., 2018). Therefore, the specific balance mechanism between microalgae and microorganisms needs to be further explored to lay the foundation for the enrichment of microbial metabolites and to improve biomass production.

**Figure 1:**
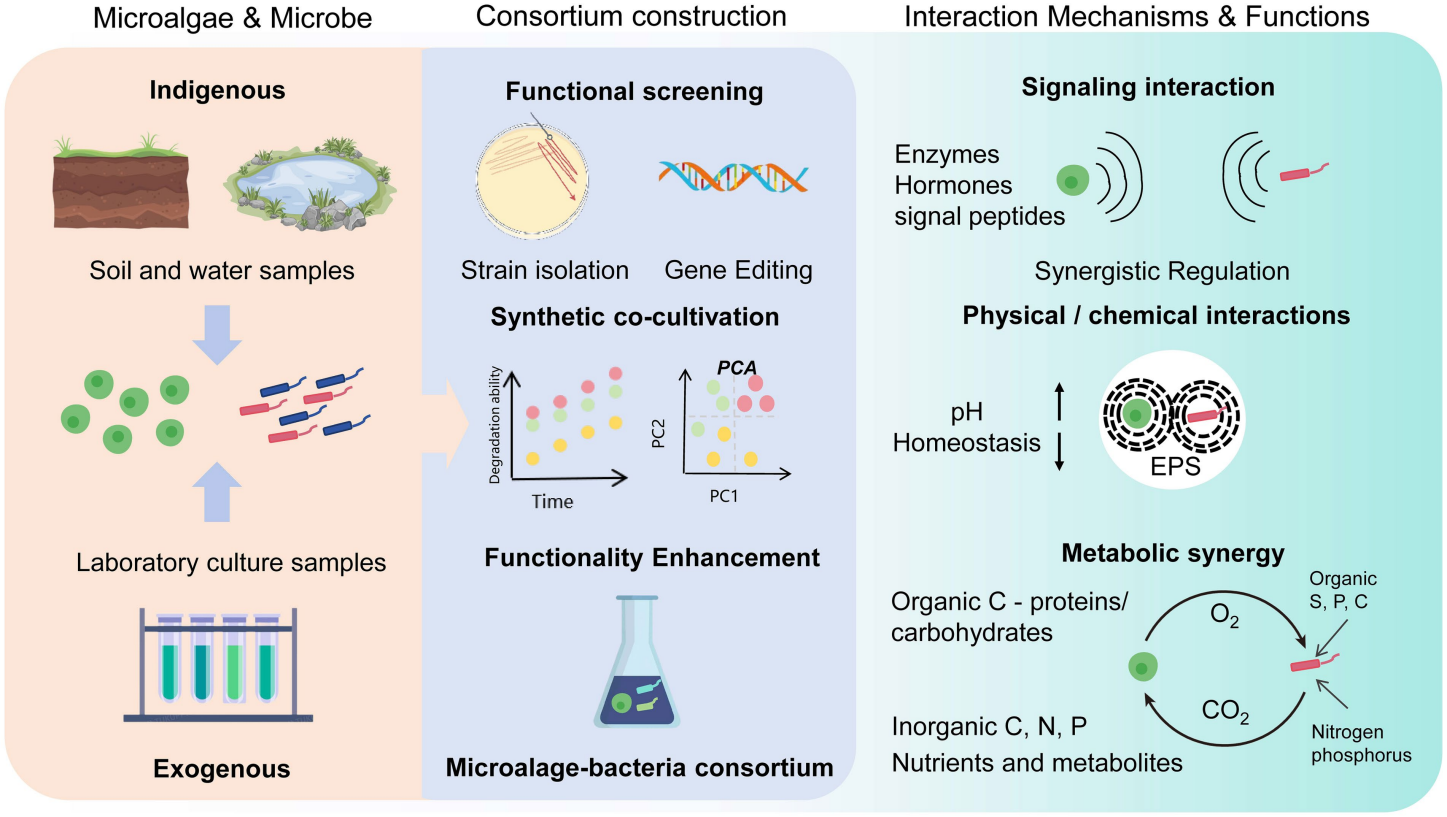
Proposed mechanisms and construction processes of microalgal–microbial symbiotic systems. Indigenous and exogenous microorganisms are distinguished by origin. Exogenous strains (e.g., algae/bacteria) are isolated via native habitat sampling, gradient dilution, selective media, and functional screening, followed by synthetic co-cultivation. Genome editing enhances stress resilience and growth-promoting traits. (right): Metabolic synergy involves algae fixing CO₂ and secreting dissolved organic carbon (DOC; e.g., glycoproteins, exopolysaccharides), while bacteria respire CO₂ and supply siderophores, phytohormones, and extracellular polymeric substances (EPS). Complementary EPS compositions (polysaccharide–protein in algae vs. lipopeptides in bacteria) stabilize biofilm matrices. pH homeostasis is achieved via algal photosynthesis (pH↑) and bacterial organic acid secretion (pH↓). Molecular crosstalk via extracellular enzymes and hormones coordinates growth and gene expression.

Figure 1 reveals the three main mechanisms by which microalgae interact with microorganisms: substrate exchange, chemical mediators, and intercellular exchange (Zhang et al., 2021a). Typically, interactions between microalgae and microorganisms occur in the form of symbiosis (Chia et al., 2023). Microalgae can attract beneficial bacteria while repelling dangerous bacteria through the production of antibiotic compounds (Yang et al., 2022). In the symbiotic consortium of microalgae and bacteria, bacteria benefit from microalgae because they produce extracellular compounds and oxygen; in exchange, microalgae obtain carbon dioxide, vitamins, and other nutrients from the bacteria (Solomon et al., 2023).

In a symbiotic system, nitrogen-fixing bacteria convert atmospheric nitrogen into inorganic nitrogen, while microalgae provide the nitrogen-fixing bacteria with an essential carbon source (Zhang et al., 2021b). For example, *Synechococcus* sp. forms a long-term symbiotic relationship with a heterotrophic bacterium. This interaction promotes the self-sustainability of the nitrogen cycle, encompassing the processes of nitrogen fixation, denitrification, and organic nitrogen degradation (Zhang et al., 2021b). As a result, the symbiotic system is able to maintain healthy growth of the cyanobacterium for up to two years, even in the absence of exogenous nutrient supply (Zhang et al., 2021b). Furthermore, because consortia of microalgae and nitrogen-fixing bacteria can perform more complex tasks than they can alone, this can be harnessed to improve biotechnological applications, such as by reducing production costs and increasing microalgal biomass yields (Llamas et al., 2023).

In addition to bacteria, fungi have the potential to form a mutually beneficial symbiotic relationship with microalgae in the form of lichens. Microalgae–fungal symbiotic relationships are usually established by introducing fungal mycelial particles into microalgal media and mixed cultures (Molins et al., 2018; Wang et al., 2023; Bonito, 2024). The surface of microalgae is negatively charged and the polysaccharides on the surface of fungi are positively charged, which causes the microalgae and fungal culture to aggregate because the microalgae become trapped in the fungal hyphae and separate from the surrounding fluid; this structure easily immobilizes microalgae (Laezza et al., 2022), which is beneficial for the fixation of the consortium in saline–alkali soil and the exertion of its beneficial effects.

Filamentous fungi, in addition to forming a symbiotic relationship with microalgae, can be used as a carrier material for immobilizing microalgae (Talukdar and Barzee, 2025). The symbiotic relationship between microalgae and yeast such as *Saccharomyces* is based on the complementary nature of nitrogen and carbon metabolism. Nitrogen is essential for synthesizing antioxidant enzymes and substances in plants, whereas carbon is the foundation for plant growth and metabolism. During fermentation, yeast produces carbon dioxide, which microalgae use for photosynthesis, thereby promoting their growth; simultaneously, the oxygen produced by microalgal photosynthesis can be used by yeast to facilitate its fermentation activities. This relationship enhances the metabolic efficiency and stability of the entire system and has been implemented as an effective method for wastewater treatment (Abdalla et al., 2024). From a biomass point of view, the reciprocal symbiosis between fungi and microalgae can increase the biomass and reduce the cost of cultivation (Wang et al., 2022). The co-cultivation of suitable microalgae and microbial members has the potential to promote plant growth or enhance stress resistance in specific environments, such as those under salt–alkali stress.

## 5 Microalgae and microorganisms synergize to promote plant growth under saline and alkali stress

**Figure 2:**
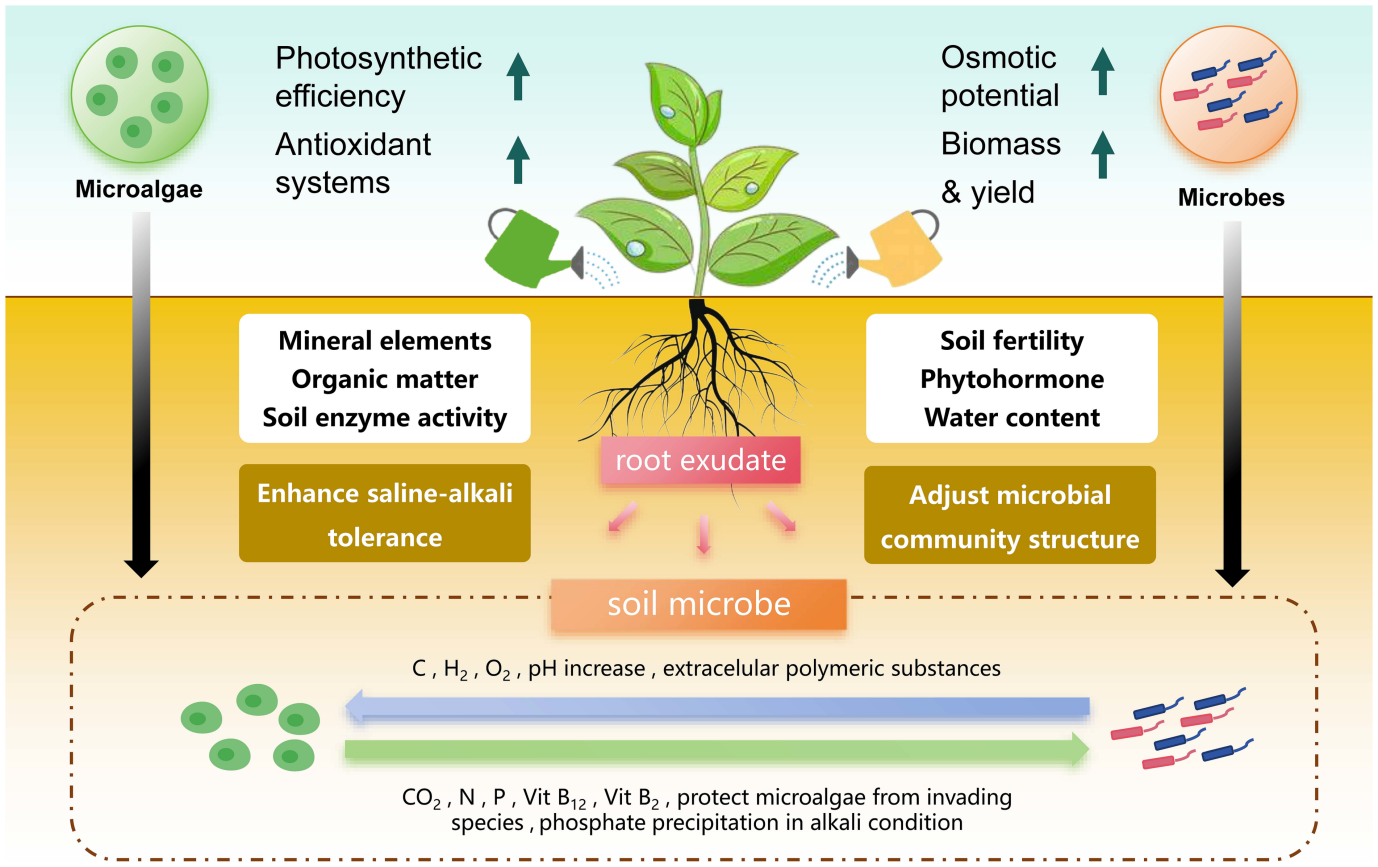
Symbiotic dynamics of microalgae and microorganisms in enhancing plant growth and saline soil adaptation. The symbiotic relationship among microalgae, soil microorganisms, and plants enhances plant salt tolerance. Microalgae supply plants with water, nutrients, and chlorophyll via photosynthesis, enhancing soil organic matter, enzyme activity, and crop yield. Microorganisms modulate soil fertility, plant hormones, and water content, and refine microbial community structure through root exudates. In this bidirectional interaction, microalgae release dissolved organic carbon (DOC), hydrogen (H₂), oxygen (O₂), and extracellular polymeric substances into the soil, which increase soil pH and promote microbial growth. In return, microorganisms produce carbon dioxide (CO₂), nitrogen (N), phosphorus (P), vitamin B12, and vitamin B2. These vitamins protect microalgae from invasion and facilitate phosphate precipitation under alkali conditions. This synergistic relationship enhances plant stress resistance and accelerates adaptation to saline–alkali environments, demonstrating the potential of soil microorganisms to improve agricultural sustainability.

In agriculture, the application of microalgae–microbial co-culture technology aims to enhance nitrogen fixation and increase phosphorus and potassium, especially under saline and alkaline conditions, and to improve environmental adaptability for plant growth (Gavilanes et al., 2020). However, the current research on the interaction between microalgae and microorganisms in agricultural environments is relatively limited (Zhou et al., 2023). By simulating the microalgae–microbial co-culture system, as shown in Figure 2, we conducted an in-depth review of the synergistic effects of microalgae and microorganisms in the soil.

As shown in Table 2, the microalgae–microbial symbiosis system has significant plant growth promotion effects in agriculture, which result in improved plant nutritional status, stress tolerance, and regulation of growth environment. Cyanobacteria provide plants with oxygen and organic matter through photosynthesis, and simultaneously convert atmospheric nitrogen into plant-available forms through nitrogen fixation. Microorganisms then act as decomposers of plants, releasing nutrients such as nitrogen, phosphorus, and potassium (Llamas et al., 2023). In particular, microorganisms such as bacteria and fungi can decompose plant cell walls in litter by secreting enzymes that release nutrients required by living plants (Schroeter et al., 2022).

**Table 2:** Examples of how microalgae and microbes work together to boost plant growth.

Plant	Microalgae	Microbes	Effect	References
Wheat	*Chlorella pyrenoidosa*	Nitrogen-fixing bacteria *Azotobacter beijerinckii*	Microalgae and microbes together were more successful in reducing salt stress and nitrogen shortage than either alone.	Zhou et al. (2023)
	*C. vulgaris* SAG 211 19	*B. subtilis* MT300403	*Bacillus subtilis* isolated from microalgae–bacteria co-cultures demonstrated tolerance under extremely harsh conditions, alleviating salinity and pH stress.	Bui-Xuan et al. (2022)
*Helianthus annuus* L.	*Leptolyngbya* sp. XZMQ	*Bacillus* XZM	Double inoculation enhanced microbial carbon and nitrogen fixation, boosting soil fertility.	Mao et al. (2024)
*Phaseolus vulgaris*	*Anabaena cylindrica*	*Rhizobium* (*R. tropici*+*R. freirei*)	Co-inoculation with Riz + Azo + Ana improved soybean plant height, root dimensions, above-ground biomass, rhizome count and dry matter at flowering, seed pod density and overall yield.	Horácio et al. (2020)
Onion (*Allium cepa* L.)	*Spirulina*	*Pseudomonas*	*S. platensis* extract and *P. stutzeri* inoculation synergistically enhanced onion growth and yield.	Geries and Elsadany (2021)
Lettuce	Fresh water algae (*Chlorella vulgaris*)	Bacteria that promote growth include *Herbaspirillum* sp., *Azotobacter* sp., *Azospirillum* sp., and *Bacillus licheniformis*	Total carotenoids, total antioxidant capacity, and lettuce output were all positively impacted by mycorrhizal treatments.	Maestre et al. (2012)
*Zea mays*	*Anabaena cylindrica* (cyanobacteria)	*Azospirillum brasilense*	Co-inoculation of *A. cylindrica* and *A. brasilense* enhanced hybrid maize growth, development, and yield compared with uninoculated *A. brasilense* controls.	Gavilanes et al. (2020)
Rice	Cyanobacterial strains CR1, CR2 and CR3 (*Anabaena* sp., *Calothrix* sp., *Anabaena* sp.)	PR3, PR7, and PR10 (*Providencia* sp., *Brevundimonas* sp., *Ochrobacterium* sp.)	PR10 (*Ochrobacterium* sp.) and CR2 and CR1 (both *Anabaena* sp.) were effective in increasing rice growth and grain output while also promoting soil health. They also contributed to the 40–80 kg/ha nitrogen savings.	Prasanna et al. (2012)
Cucumber	Cyanobacteria	*Azotobacter* sp.	As a measure of growth, photosynthetic pigments increased two to three times, and defense and antioxidant enzyme activity were triggered. There was significant improvement of plants in the infected treatments.	Simranjit et al. (2019)

This symbiotic system improves plant salt tolerance by promoting antioxidant enzyme activity, ion homeostasis control, and synthesis of osmoregulatory compounds. Additionally, it strengthens the plant antioxidant system by reducing soil salt concentration through photosynthesis (Pei and Yu, 2023). Some other ways by which a plant’s antioxidant defense can be enhanced by microalgae–bacteria consortia include increased production of bioactive compounds such as carotenoids, activation of defense enzymes, ascorbate system modulation that mitigates oxidative stress, phenylpropanoid pathway stimulation that increases reactive oxygen species scavenging, and synergistic nutrient exchange that enhances stress tolerance (Kang et al., 2021; Abate et al., 2024). Strengthening of the plant antioxidant system can consequently improve tolerance to oxidative stress (Zhou et al., 2023). For instance, co-culture of *Chlorella vulgaris* with PGPB that was applied to lettuce was found to increase total carotenoid content and plant weight under stress conditions (Kopta et al., 2018).

In addition, microalgae and microorganisms in the symbiotic system interact with each other to promote the production of beneficial compounds such as phytohormones, amino acids, and polysaccharides, which enhance plant resistance to salt stress (Hristozkova et al., 2018; Maurya et al., 2024). For example, G-protein-coupled receptor (GPCR)-mediated signal transduction is a pathway used by most bacteria and cyanobacteria in the phyllosphere and rhizosphere to detect and respond to salt stress (Canfora et al., 2014). Key microbes containing GPCRs and G-proteins produce compatible solutes like glycine betaine, sucrose, and glucosylglycerol under salt stress; these solutes bind to GPCRs and trigger downstream signaling pathways that help mitigate the effects of salt stress (Canfora et al., 2014). Furthermore, *Azotobacter beijerinckii* and *C. pyrenoidosa* were found to provide each other with hormones, carbon dioxide, and organic matter for growth, demonstrating the critical role of their symbiosis in plant growth and stress tolerance (Chia et al., 2021).

Symbiotic systems can also improve the soil environment and mitigate the negative effects of salinity on plants, such as by increasing soil organic matter, improving crop growth, nutrient mobilization, and nutrient status, and increasing water retention capacity (Prasanna et al., 2012). In contaminated soils, symbiotic systems are involved in bioremediation, restoring soil function by degrading organic pollutants and absorbing heavy metals. For example, the combined application of *Bacillus* and *Chlorella* improved rice soil pH, promoted soil microbial activity, increased soil phosphorus, nitrogen, and organic carbon content, and improved soil quality (Prasanna et al., 2012).

Symbiotic microalgae and bacteria regulate plant–soil microbial interactions and improve plant salt tolerance by influencing the structure and function of soil microbial communities (Zhou et al., 2023). Plant salt tolerance can be improved by microbial communities by induction of leaf senescence as a result of protein synthesis and inhibiting photosynthesis in response to accumulation of reactive oxygen species, which occurs under high salinity, and decreasing photosynthesis in response to salt stress (Maurya et al., 2024). A study showed that the combined application of *Bacillus megaterium* and *C. vulgaris* significantly increased the metabolic activity and proliferation rate of the soil microbial community, and increased the relative abundance of *B. megaterium* and *C. vulgaris* in the soil (Dao et al., 2018). Thus, microalgae–microbial symbiosis provides an important strategy for sustainable agriculture by modifying the soil microbial community and promoting plant growth under salt stress, providing a new strategy for sustainable agricultural development.

### 5.1 Exogenous microalgae promote plant growth by altering soil microbial communities

A mutually beneficial symbiotic relationship may be formed between microalgae and soil-originating microorganisms, in which the microalgae provide oxygen, organic matter, and phytohormones (Gonzalez-Gonzalez and De-Bashan, 2023), while the microorganisms decompose the organic matter to release nutrients for the microalgae to utilize; together, they promote the stability of the soil ecosystem. However, the extent to which exogenous microalgae affect soil microbial communities is influenced by a variety of factors, including microalgae species, the amount of microalgae added, soil type, and climatic conditions (Maity et al., 2014; Gonçalves et al., 2023). For example, different microalgae species such as *Chlorella* (Ma et al., 2023), *Spirulina* (Bahmani Jafarlou et al., 2021), *Scytonema hofmanni* (Rodríguez et al., 2006), and *Chlamydomonas* (Calatrava et al., 2023) have varying effects on soil microbial communities, and increased microalgae additions may lead to increased nutrient competition among soil microbes.

In addition, soil moisture, salinity conditions, and climatic factors affect the growth and metabolism of microalgae, which in turn affects the soil microbial community. Studies have shown that the combination of microalgae with PGPB, such as *Bacillus subtilis*, can significantly increase the activity of carbon and nitrogen-fixing microorganisms in the soil, thereby improving soil fertility (Setubal et al., 2009; Renuka et al., 2018). Exogenous cyanobacterial treatments have also been shown to improve soil fertility and promote crop growth, development, and yields (Prasanna et al., 2009; Nain et al., 2010).

Furthermore, the microalgae *Chlamydomonas* has emerged as a promising candidate for bioremediation and biofuel production (Banerjee et al., 2021; Bellido-Pedraza et al., 2024; Torres et al., 2024). *Chlamydomonas* employs several mechanisms to remove pollutants from wastewater, such as biosorption, in which the cell wall, rich in various chemical groups, acts as a sorbent to capture contaminants (Leong and Chang, 2020); bioaccumulation, in which pollutants are taken up and stored within *Chlamydomonas* (Hoyos et al., 2023); and biotransformation, in which pollutants are broken down through enzymatic processes into simpler, less toxic compounds (Touliabah et al., 2022). *Chlamydomonas* has shown significant potential as a biofertilizer because of its rich nutrient profile and ability to enhance soil properties and plant growth. It can improve soil structure and stability (Metting, 1986) and organic matter and microbial activity (Miranda et al., 2024); it has biostimulant properties, such as auxin-like activity, which can increase root development and improve overall plant health (Stirk et al., 2002); it enhances nutrient uptake of plants, which can improve growth and yield (Gitau et al., 2021; Martini et al., 2021); it reduces the need for chemical fertilizers that may have negative environmental impacts (Miranda et al., 2024); and its production can be cost effective if integrated with wastewater treatment (Sido et al., 2022). Given the multifunctional capabilities of *Chlamydomonas* in pollutant removal, soil improvement, and plant growth promotion, it holds significant potential for application in saline–alkali soil remediation and enhancing plant salt tolerance. Therefore, more research is needed on how *Chlamydomonas* affects plant growth under saline stress.

However, the introduced exogenous microalgae may also compete with native soil microorganisms for resources, triggering competition that leads to a reduction in the size of native microbial populations or changes in ecological niches (Xie et al., 2025), ultimately disrupting the natural balance of the soil ecosystem. In agricultural practices, the use of exogenous microalgae or bioinoculants can also raise other concerns, such as risks of introducing pathogens or harmful substances that could affect plant health, soil quality, and potentially human health if they enter the food chain, and unintended consequences, such as changes in soil chemistry or the release of substances harmful to other organisms in the ecosystem (García et al., 2017; Yu et al., 2024).

Currently, relatively few studies on the effects of exogenous microalgae on soil native microorganisms have been conducted under laboratory simulation conditions, and the complexity and diversity of soil ecosystems make it challenging to predict and quantify the effects of exogenous microalgae on native microorganisms. To more fully assess the effects of microalgae application in agro-soil ecosystems, future studies need to consider multiple factors such as soil type, climatic conditions, and vegetation type.

### 5.2 Exogenous microorganisms can promote plant growth by enhancing microalgal soil communities

The effect of exogenous microorganisms on soil microalgal communities is a complex process involving the interaction of multiple factors, and its effect is influenced by the microbial species, their number, and the characteristics of the microalgal communities. A study showed that inoculation of salt-tolerant *Bacillus shortus* (STR2), salt-tolerant *Aeromonas* (STR8), and oxidation-tolerant *Bacillus exotica* (STR36) had a significant effect on the microbial community associated with maize roots, with salinity and inoculation of plant growth-promoting rhizobacteria being the key factors. Inoculation of salt-tolerant rhizobacteria helped to maintain the stability of the microbial community structure and enhance its resilience, which may indirectly affect the abundance of soil microalgal populations (Bharti et al., 2015).

As a result of the benefits of microalgae–microorganism symbiosis (e.g., providing nutrients to microalgae, promoting nutrient cycling, and alleviating environmental stress), inoculation of soil with functional microorganisms can recruit and stimulate other microalgae or rhizosphere microorganisms to play an active role in helping heterotrophic microorganisms grow rapidly and increasing their metabolic activity (Papin et al., 2025). Nitrogen-fixing bacteria such as *Pseudomonas aeruginosa* play an active role in the development of maize plants through the expression of the nitrogen-fixing enzyme gene nifH, and the maize population increases under co-inoculated conditions (Ke et al., 2019).

Microalgae–bacteria symbiotic systems increase soil fertility and control inter-root microalgae by altering the abundance and composition of soil microorganisms and enzymes, soil fertility, and controlling the composition and activity of inter-root soil microbial populations. However, the introduction of exogenous microorganisms may trigger competition for resources with native soil microorganisms or the secretion of inhibitory substances that can negatively affect plant growth (Solomon et al., 2024). Although plant growth-promoting microorganisms play a key role in soil ecosystems, participating in processes such as nutrient cycling, organic matter decomposition, and increasing plant resistance to saline and alkali stress, there is a lack of clarity about (1) how added microorganisms help plants cope with the negative impacts of changes in the microbial community and (2) the types of microorganisms that should be added and the criteria for doing so.

Specific cyanobacterial species, such as *Anabaena* and *Nostoc*, can significantly enhance plant growth and yield by fixing atmospheric nitrogen, thereby increasing soil nitrogen availability (Renuka et al., 2018). For example, cyanobacterial inoculants can improve the growth and yield of crops such as maize (Prasanna et al., 2016). Additionally, cyanobacteria secrete polysaccharides and proteins that contribute to soil aggregation and stability, and research has demonstrated that the application of specific cyanobacterial inoculants can significantly increase the levels of glomalin-related soil proteins and polysaccharides, which are crucial for soil structure (Prasanna et al., 2016). Cyanobacterial inoculants can also induce the production of defense enzymes in plants, such as phenylalanine ammonia-lyase and polyphenol oxidase, thereby enhancing resistance to pathogens (Prasanna et al., 2015), and cyanobacterial inoculants are eco-friendly and cost-effective alternatives to chemical fertilizers. Therefore, they may help reduce reliance on chemical inputs while maintaining soil biodiversity and health (Prasanna et al., 2016).

However, there are also limitations associated with using particular microalgae. There can be variable effectiveness of cyanobacterial inoculants, which can be influenced by soil type, climate conditions, and plant variety. This variability may limit their consistent application across different agro-ecological systems (Prasanna et al., 2016). The cultivation and application of cyanobacteria require specific technical conditions, including appropriate culture media, temperature, and light conditions, which may pose challenges for widespread adoption in some regions (Prasanna et al., 2016). Furthermore, their overgrowth can lead to ecological issues such as algal blooms. Thus, careful assessment of ecological risks is necessary when using cyanobacterial inoculants. Finally, while cyanobacteria can improve soil fertility, completely replacing chemical fertilizers with cyanobacterial inoculants may not be feasible in high-yield agricultural systems, and they are more likely to serve as a supplement to chemical fertilizers (Prasanna et al., 2016).

Additionally, there are some drawbacks and challenges that need to be considered. In complex microbial communities, different microbial species may compete, which can affect their synergistic effects. For example, certain bacteria and algae may compete for nutrients, thereby reducing the overall effectiveness of biofertilizers (Mutum et al., 2022). Microorganisms may also exhibit different activities under various environmental conditions, such as soil pH, moisture, and temperature; certain algae and bacteria may show good synergistic effects under specific soil conditions but perform poorly under others (Prasanna et al., 2012). Moreover, the long-term survival and activity of microorganisms in soil may be affected by various factors, including changes in the soil microbial community and environmental stress, and some microorganisms may show good effects in the short term, but their activity may decline over time (Ramakrishnan et al., 2023).

Allelopathic interactions and autotoxicity should also be considered. Different microbial species may compete for limited resources such as nutrients, water, and space. For example, some bacteria and fungi may produce allelochemicals that inhibit the growth of other microorganisms to gain a competitive advantage (Lal and Biswas, 2023). Allelopathy can change the composition and structure of microbial communities (Weidenhamer et al., 2023) and affect plant growth by inhibiting seed germination, root growth, and plant nutrient uptake (Lal and Biswas, 2023). Some microorganisms may produce allelochemicals that not only affect other species but also inhibit their own growth; this autotoxicity can limit the population density and growth of the microorganisms themselves (Lal and Biswas, 2023).

Genetic engineering and synthetic biology approaches offer powerful tools to enhance production in agriculture and bioremediation by optimizing plant–microbe interactions. These strategies can lead to more sustainable and efficient systems for food production and environmental cleanup (Basu et al., 2018; Ke et al., 2021). Synthetic microbial communities (SynComs) technology can be utilized to design microorganisms with genetically defined properties (Tang, 2019), such as microbes that can tolerate high-salt environments, suppress disease, and help degrade harmful substances in the soil of the native habitat (Pradhan et al., 2022; Schmitz et al., 2022; Zhou et al., 2022; Bu et al., 2024). Although SynComs technology is in development and still being optimized, microbial community construction may effectively reduce antagonistic interactions, achieve optimal strain ratios, and address existing drawbacks and challenges.

## 6 Conclusion

This review suggests that a high degree of integration of microalgae and microorganisms can promote sustainable development under saline agriculture. This concept has the following obvious advantages: (1) the metabolic complementarity between microalgae and microorganisms can further enhance the salt tolerance of plants and help to increase the overall growth rate of the symbiotic system; (2) the diversity of microalgae–microorganism symbiotic system can help to improve the stability and resistance of the whole ecosystem and reduce the incidence of plant diseases; (3) the microalgae–microbial symbiosis system can produce bioactive substances to promote plant growth, such as phytohormones, which can help to improve the seedling rate, survival rate and biomass of crops; and (4) microalgae–microbial symbiosis can improve the structure of the soil, increase the content of organic matter, and improve the water storage capacity and fertility of the soil.

The interactions between microalgae and bacteria are highly significant in the context of the circular economy, particularly for biomass derived from wastewater remediation that can be utilized as a biostimulant. These interactions enhance the efficiency of wastewater treatment and the productivity of biomass, which can subsequently be used for various purposes, such as bioenergy production or as a biostimulant to promote plant growth (Li et al., 2018; Rodrigues de Assis et al., 2020). The circular economy approach using microalgae–bacteria consortia can also reduce production costs and minimize the release of pollutants into the environment; treated effluent can be safely discharged or used for irrigation, while the biomass can be converted into biofuel or biofertilizers, reducing waste and promoting sustainable practices (Rodrigues de Assis et al., 2020).

## 7 Future perspectives

Microalgae–microbial symbiotic systems, although a key strategy in saline agriculture, also face several challenges in their research and application. The high cost of microalgae cultivation and application is the main factor limiting its wide application in agriculture (Zhao et al., 2019; Song et al., 2022). Therefore, reducing cost and increasing efficiency are essential. However, scaling up laboratory-scale microalgae culture technology to large-scale production in native soils requires solving a series of technical problems, including light intensity distribution, nutrient supply, and pest and disease control.

To make the process of using microalgae–bacteria consortia economically viable for biostimulant or agricultural applications, strategies need to be employed to efficiently obtain the necessary biomass, such as by using high-density cultivation techniques and using techniques to reduce harvesting costs (Menegazzo and Fonseca, 2019; Jin et al., 2021), and determine the most cost-effective sources of nutrients (Kumar et al., 2020; Kuo et al., 2021), such as by obtaining organic carbon sources from lignocellulosic biomass or industrial waste products and implementing nutrient recycling strategies (Chia et al., 2023).

To optimize the efficiency of these systems, species selection of microalgae and microorganisms should focus on screening and cultivation of saline-tolerant species to improve the efficient utilization of both in saline soils and to promote plant growth and development in saline environments. Genetic engineering and SynComs technology offer substantial opportunities to enhance agriculture by designing microorganisms that help plants better absorb water and nutrients, thus improving their salt tolerance, or that promote soil nutrient cycling, which can reduce chemical fertilizer use, improve soil structure, and increase soil fertility (Basu et al., 2018; Ke et al., 2021; Srikanth et al., 2025).

Despite recent advancements, critical knowledge gaps persist in optimizing microalgae–microbial systems for saline agriculture. For instance, the long-term ecological impacts of introducing engineered microbial consortia into saline soils remain underexplored, particularly regarding their interactions with native soil microbiomes and ecosystem stability. Additionally, there is limited understanding of how environmental variables (e.g., fluctuating salinity, temperature, and light regimes) influence the functional resilience of these systems under field conditions. Further studies are needed to elucidate the metabolic crosstalk between microalgae and specific microbial taxa, and to develop predictive models for scaling up co-culture systems while maintaining cost-effectiveness and sustainability. Bridging these gaps will require interdisciplinary approaches integrating omics technologies, advanced bioreactor design, and real-world field trials to translate laboratory findings into practical agricultural solutions.
